# The heterogeneity of the COVID-19 pandemic and national responses: an explanatory mixed-methods study

**DOI:** 10.1186/s12889-021-10885-8

**Published:** 2021-05-01

**Authors:** Yi-Ying Chen, Yibeltal Assefa

**Affiliations:** School of Public Health, the University of Queensland, Brisbane, Australia

**Keywords:** COVID-19 pandemic response, Australia, Brazil, China, Germany, Italy, New Zealand, South Korea, Thailand, United States

## Abstract

**Background:**

The coronavirus disease of 2019 (COVID-19) has quickly spread to all corners of the world since its emergence in Wuhan, China in December of 2019. The disease burden has been heterogeneous across regions of the world, with Americas leading in cumulative cases and deaths, followed by Europe, Southeast Asia, Eastern Mediterranean, Africa and Western Pacific. Initial responses to COVID-19 also varied between governments, ranging from proactive containment to delayed intervention. Understanding these variabilities allow high burden countries to learn from low burden countries on ways to create more sustainable response plans in the future.

**Methods:**

This study used a mixed-methods approach to perform cross-country comparisons of pandemic responses in the United States (US), Brazil, Germany, Australia, South Korea, Thailand, New Zealand, Italy and China. These countries were selected based on their income level, relative COVID-19 burden and geographic location. To rationalize the epidemiological variability, a list of 14 indicators was established to assess the countries’ preparedness, actual response, and socioeconomic and demographic profile in the context of COVID-19.

**Results:**

As of 1 April 2021, the US had the highest cases per million out of the nine countries, followed by Brazil, Italy, Germany, South Korea, Australia, New Zealand, Thailand and China. Meanwhile, Italy ranked first out of the nine countries’ total deaths per million, followed by the US, Brazil, Germany, Australia, South Korea, New Zealand, China and Thailand. The epidemiological differences between these countries could be explained by nine indicators, and they were 1) leadership, governance and coordination of response, 2) communication, 3) community engagement, 4) multisectoral actions, 5) public health capacity, 6) universal health coverage, 7) medical services and hospital capacity, 8) demography and 9) burden of non-communicable diseases.

**Conclusion:**

The COVID-19 pandemic manifests varied outcomes due to differences in countries’ vulnerability, preparedness and response. Our study rationalizes why South Korea, New Zealand, Thailand, Australia and China performed better than the US, Italy and Brazil. By identifying the strengths of low burden countries and weaknesses of hotspot countries, we elucidate factors constituting an effective pandemic response that can be adopted by leaders in preparation for re-emerging public health threats.

## Background

The coronavirus disease of 2019 (COVID-19) was first reported in Wuhan city of the Hubei Province in China around late December 2019 [1]. It was not until one month after the initial outbreak that the World Health Organization (WHO) declared the situation as a global health emergency [2]. After over one year, the virus has been traced in every corner across the globe, surpassing 130 million total confirmed cases and 2.8 million total confirmed deaths as of 4 April 2021 [3].

The burden of COVID-19 has been heterogeneous across regions of the world: Americas (56,589,190 cumulative cases and 1,368,633 deaths), Europe (45,877,941 cumulative cases and 980,586 deaths), Southeast Asia (15,212,235 cumulative cases and 222,054 deaths), Eastern Mediterranean (7,693,094 cumulative cases and 160,612 deaths), Africa (3,120,296 cumulative cases and 78,523 deaths), Western Pacific (1,965,683 cumulative cases and 31,904 deaths) [3]. Overall, Americas has the largest cumulative number of cases, followed by Europe, South-East Asia, Eastern Mediterranean and Africa. The Western Pacific region has the least epidemiological epidemic [3]. This has revealed inequalities in healthcare access and inadequacies in health infrastructures, even amongst countries ranked highest in preparedness on the Global Health Security Index [4].

Initial outbreak responses varied greatly between governments, ranging from proactive and comprehensive in some to delayed and disorderly in others. While many existing studies strive to understand and reflect on how individual countries could have handled the pandemic differently, there is a lack of research that systematically compares and contrasts pandemic responses in different parts of the world. In this paper, we aim to fill the knowledge gap by understanding the strengths of low burden countries and weaknesses of hotspot countries. We will consider nine action areas, which can be adopted by leaders to develop more effective pandemic response plans in preparation for future public health threats.

## Methods

### Research Design

This study was conducted through a mixed-methods approach that integrated quantitative and qualitative secondary data. The following steps were included in the research process: (i) collecting quantitative epidemiological data, (ii) methodically selecting nine countries to be included in the study, (iii) conducting literature review of qualitative secondary data and (iv) systematically evaluating countries’ pandemic responses to explain the variability in their COVID-19 epidemiological outcomes. Through this procedure, our study elucidates specific factors that constitute an effective and sustainable pandemic response.

### Country Selection

Nine countries were selected for this study, and they were chosen based on three factors: income level, relative COVID-19 outcome and geographic region. First, selection was limited to high-income and upper-middle-income countries, where more cases and deaths have been recorded due to the prevalence of air travel that contributed to early onset and widespread transmission of COVID-19 [5]. The observed difference in case number can also be explained by the fact that wealthier countries have more sufficient resources dedicated towards testing than do lower-middle-income and low-income countries [6].

Of all the high-income and upper-middle-income countries, we narrowed the list down to the United States (US), Italy, Germany, Australia, South Korea, New Zealand, Brazil, China and Thailand. These countries not only offered geographic and demographic diversity, but also reflected stark contrasts in pandemic outcomes. For the purposes of this study, we developed a framework to categorize those countries into high, medium versus low COVID-19 burden. The framework involved inputting total cases per million and total deaths per million for all high-income and upper-middle-income countries onto an Excel Spreadsheet. The first and third tertiles were computed and used as the cut-off values between burden levels. Based on the calculation, “low burden countries” had fewer than 17,444.687 total cases per million and fewer than 287.438 total deaths per million as of 1 April 2021. “High burden countries” had over 52,564.861 total cases per million and over 955.338 total deaths per million as of 1 April 2021. “Medium burden countries” measured between the two aforementioned groups.

Every high, medium and low burden country offers a unique perspective and learning opportunity for pandemic management. China was the first country to report a COVID-19 outbreak. Through intensive control strategies, the nation recovered from its initial case spike and was included in our study as a low burden country. In the months following, uncontainable outbreaks occurred across North and South America in high burden countries like the US and Brazil. In Europe, several European Union (EU) members linked their first COVID-19 cases to the initial outbreak in Italy, another highly devastated country in our study. Among the impacted EU members, Germany was chosen as a medium burden country, having curbed its fatality rate through a robust hospital capacity. Meanwhile, Australia was selected as a low burden country that effectively flattened the curve via mass testing protocols. Also included in the study is New Zealand, which was chosen for its top performance since the start of the pandemic. Finally, South Korea and Thailand were both selected for their geographic proximity to China and paradoxically low disease burden.

### Indicators

To explain the clinical variability between the nine countries, a list of 14 indicators was established to systematically assess each country’s preparedness, actual pandemic response, and overall socioeconomic and demographic profile in the context of COVID-19. The 14 indicators used in this study include: 1) Universal Health Coverage Index, 2) public health capacity, 3) Global Health Security Index, 4) International Health Regulation and Joint External Evaluation, 5) leadership, governance and coordination of response, 6) community mobilization and engagement, 7) communication, 8) testing, quarantines and social distancing guidelines, 9) medical services at primary health care facilities and hospitals, 10) multisectoral actions and continuity of essential services, 11) social protection services, 12) absolute and relative poverty status, 13) demography, and 14) burden of communicable and non-communicable diseases. Comparing and contrasting the chosen countries through these multifaceted lenses bring forth a better understanding of what is entailed in a successful pandemic response. Moreover, systematically evaluating these indicators allows us to rationalize why some countries have high disease burden, while other countries are faring better under the pandemic.

### Data Sources

This study incorporated quantitative and qualitative secondary data that are publicly available. First, we used the “GNI per capita, Atlas method (current US$)” dataset from the *World Bank* website to categorize countries into high-income, upper-middle-income, lower-middle-income and low-income statuses [7]. For the purposes of this study, we focused on high-income and upper-middle-income countries, which have gross national income (GNI) above $4045 per capita [8]. Secondly, we accessed epidemiological data on confirmed cases, cases per million, confirmed deaths, deaths per million, testing per 1000, age structures and non-communicable disease (NCD) disability-adjusted life years (DALYs) through the *Our World in Data* website’s datasets [9, 10]. Using the confirmed cases and deaths data, we then calculated the case fatality rates (CFRs) for the nine countries.

To systematically evaluate countries based on the 14 aforementioned indicators, exploratory qualitative data analysis was performed. In this study, qualitative secondary data was obtained from peer-reviewed journals, reputable online news outlets, government reports and publications by public health-related associations, such as the WHO.

## Results

### Epidemiological Findings

Table 1 displays the COVID-19 epidemiological data and groups the nine countries based on their income status and relative COVID-19 burden level. As of 1 April 2021, the US had the highest cases per million out of the nine countries, followed by Brazil, Italy, Germany, South Korea, Australia, New Zealand, Thailand and China. Italy ranked first out of the nine countries’ total deaths per million, followed by the US, Brazil, Germany, Australia, South Korea, New Zealand, China and Thailand. In terms of CFR, which is the ratio of deaths to confirmed cases, China had the highest value, followed by Australia, Italy, Germany, Brazil, the US, South Korea, New Zealand and Thailand. Testing data was not uniformly updated in all nine countries. However, the most updated record as of 1 April 2021 showed that the US had the highest total tests per 1000 population, followed by Italy, Australia, Germany, New Zealand, South Korea, China, Thailand and Brazil [9]. The proportion of the population over age 65 and NCD DALYs will be discussed in more depth in the “Age Structure and Burden of Non-Communicable Diseases” subheading.

**Table 1:** COVID-19 epidemiological data as of *December 1st, 2020, population proportion aged 65+ and NCD DALYs in 2017 from the US, Italy, Germany, Australia, South Korea, New Zealand, Brazil, China and Thailand [7, 9, 10]

Income	High						Upper-Middle		
Burden	High		Medium	Low			High	Low	
**Country**	**US**	**Italy**	**Germany**	**South Korea**	**Australia**	**New Zealand**	**Brazil**	**China**	**Thailand**
**Total Cases**	30,539,868	3,607,083	2,866,323	104,194	29,333	2501	12,839,844	101,753	28,889
**Total Cases per Million**	92,264.72	59,658.85	34,210.89	2032.293	1150.319	518.639	60,405.91	70.695	413.883
**Total Deaths**	553,345	109,847	76,823	1737	909	26	325,284	4841	94
**Total Deaths per Million**	1671.724	1816.799	916.918	33.88	35.647	5.392	1530.32	3.363	1.347
**CFR (%)**	1.811878	3.045314	2.680193	1.667083	3.098899	1.039584	2.533395	4.757599	0.325383
**Total Tests per 1000**	1152.703	831.247	600.841 (*as of 28 Mar 2021)	149.771	615.645	394.613	30.21 (*as of 19 Sep 2020)	111.163 (*as of 6 Aug 2020)	47.301
**Population Proportion Aged 65+**	15.413	23.021	21.453	15.504	13.914	15.322	8.552	10.641	11.373
**NCD DALYs (years lost per 100,000 people)**	19,742.58	14,506.89	16,560.72	13,333.71	15,280.05	16,037.43	19,290.94	17,348.12	16,135.48

### COVID-19 Cases and Cases per Million

There were five indicators that helped explain the variability of COVID-19 cases and cases per million statistics in the nine countries. They were: 1) leadership, governance and coordination of response, 2) communication, 3) community engagement, 4) multisectoral actions and 5) public health capacity. Effective use of these action areas constituted a successful pandemic response that yielded relatively low caseload.

#### Leadership, Governance and Coordination of Response

Governments across the world have managed the COVID-19 pandemic on different timelines due to contrasting health infrastructures, economic challenges and political motivations. At the epicenter, China implemented one of the most “ambitious, agile and aggressive disease containment efforts” in its history [11]. After news about COVID-19 became public, the government increased funding towards public health services and enforced strict guidelines that prevented the gathering and movement of crowds [11]. Controversies were raised regarding the leadership’s repression of whistleblowers, who tried to share critical information about the virus in December of 2019, in the interest of maintaining a favorable public image [12]. Nonetheless, the Chinese government’s ability to bring an otherwise rampant disease under control in a population of over 1.3 billion people deserves recognition and provides important lessons for the global response.

In New Zealand, Prime Minister Ardern has been praised for setting the “gold standard” of pandemic management [13]. Her proactivity to translate epidemiological principles into tangible actions freed New Zealand of community transmission for over 100 days and placed the nation at the top of the COVID Performance Index ranking [14, 15]. In South Korea and Thailand, leaders also responded with swift communication between the central and local governments. Both countries were victims of the 2003 Severe Acute Respiratory Syndrome (SARS) and the 2015 Middle East Respiratory Syndrome (MERS) epidemics [16, 17]. Having learned from past flaws, the South Korean administration increased the number of epidemiologists and built hospitals dedicated to treating patients with infectious diseases [16, 18]. By the time the first COVID-19 case was confirmed, the government was already equipped to activate the Emergency Use Authorization (EUA) system that accelerated test kit production. Comparably, the Thai government also expanded its health workforce to include over 1000 local surveillance teams that conducted outbreak investigation, contact tracing and testing across the country. The administration has been strict on mask-wearing in public spaces, physical distancing and hand hygiene since the pandemic’s beginning [17].

Aside from having early interventions, other political leaders have highlighted the importance of putting trust in science during health crises. Chancellor Angela Merkel, for instance, embodied this quality as she led the German administration through this pandemic. As a scientist by training, she makes decisions based on objectivity and pragmatism [19]. Similarly, Australia’s relative success in managing COVID-19 was attributed to its leaders’ inclination to trust public health experts. Many politicians pushed aside political conflict and diverted full attention towards the pandemic [20]. Premiers worked together to enforce interstate traveling restrictions that minimized cross-state transmissions, resulting in a seamless, well-coordinated national response [21].

By contrast, Italy’s response was characterized as a series of incohesive regional lockdowns [22]. The government’s original intention was to only close off northern Italy for four weeks. This selective lockdown prompted a massive retreat of people toward southern Italy, inadvertently promoting exponential spread to otherwise virus-free parts of the country [22]. In a similar way, countries in Americas also saw their cases skyrocket by following the virus’s spread rather than preventing it early on. Leaders from the US and Brazil, in particular, reacted to the pandemic with a laissez-faire attitude to keep the economy afloat [23, 24]. Both countries had disorderly mask-wearing and reopening guidelines due to a lack of hierarchy between the federal and state levels. In Brazil, President Bolsonaro encouraged large public gatherings, interacted with citizens without a mask, and promoted the anti-malarial medication, hydroxychloroquine, as an effective COVID-19 treatment despite the lack of scientific backing [25]. Likewise, President Trump endorsed American healthcare facilities to stock up on hydroxychloroquine and was not seen wearing a mask in public until 11 July 2020 [26, 27]. Rather than discouraging social gatherings, he held large-scale rallies for his presidential campaign that were projected to have resulted in over 30,000 additional cases [28]. With limited experience in pandemic management, both leaders responded to COVID-19 with a denialistic attitude that not only raised people’s distrust of scientists, but also further divided the nations [29].

#### Communication

Civilians are more likely to have confidence in leaders that communicate frequently and transparently, as was seen in South Korea, Thailand and New Zealand. These three governments extensively delivered coronavirus briefings and social media campaigns since January 2020. Their daily news broadcasts invited health experts to inform the public of surveillance summaries and fact-based intervention plans [14, 16, 17]. In diverse countries like South Korea and Thailand, multilingual interpretations of COVID-19-related information were offered to enhance everyone’s understanding of personal hygiene measures and epidemiological statistics [16, 17]. Administrations also strictly prohibited the propagation of misinformation, which were tracked down immediately and addressed at news briefings [16, 17].

In New Zealand, Prime Minister Ardern was commended for conveying “clear consistent messages in an empathetic manner,” which instilled confidence and reassurance [14]. Likewise, South Korean and Thai leaders strived to reduce the public’s concern through pandemic and mental health-related call centers [16, 17, 30]. In Germany, the Robert Koch Institute (RKI) regularly published scientific data to engage with the broader community [31]. A podcast called the “Das Coronavirus-Update” also shared research-based ideas with millions of Germans through an easily consumable medium [32].

Besides television and social media outlets, computer software advancements served an integral role in the circulation of COVID-19 alerts. For instance, the South Korean government used big data to share information about the travel history of confirmed cases through smartphone safety notifications [16]. The South Korean and Thai governments both utilized mobile platforms to monitor symptoms of individuals under the compulsory 14-day quarantine [17]. In Germany, the RKI introduced the “Corona-Warn-App,” a digital contact tracing software that contributed to the nation’s successful response relative to other EU countries early on [33]. In New Zealand, the process of following-up with exposed individuals was made easier through a similar mobile application called “NZ Covid Tracer,” which helped users keep track of their own whereabouts [34].

Contrastingly, the American government showed a lack of clarity during the early stages of the pandemic. In March of 2020, the president conducted daily press conferences and used his social media platforms to lower the perceived risk of the virus. His optimistic stance diverged from those of infectious disease experts and public health officials [35]. There was also circulation of rumors about anti-malarial medicines and their impact on COVID-19 susceptibility, which stirred up false expectation amongst Americans [36]. Similar weaknesses were identified from Italy’s outbreak communication, in which a “fracture between official communications, fake news and local reactions” on mask-wearing policies generated considerable confusion [37]. Concurrently, Brazil had its own political infighting that left the population polarized and apprehensive. Publication of epidemiological reports gradually ceased, as the Brazilian president continued to dissuade people from taking the vaccine when one becomes available [38, 39]. Furthermore, mayors that attempted to follow proper health measures were regarded as political oppositions by the president [24].

While countries like Italy and Brazil lacked regulation over the circulation of false information, China sought control of its narrative about how COVID-19 originated and spread since the beginning. In December of 2019, Dr. Li Wenliang was one of the first whistleblowers to share information about patients falling ill with symptoms similar to SARS from 2003 [40]. He was quickly detained by local authorities for propagating “false rumors” and disrupting the social order [40]. Dr. Li’s silencing sparked backlash from citizens, many of whom expressed frustration at the way information was communicated and suppressed in China [40]. On the other side of the world, American healthcare workers faced similar pressure from local governments to refrain from speaking up about personal protective equipment (PPE) shortage and their fear of being in dangerous working conditions [41]. In summary, it is evident that the government’s ability to sustain open and truthful two-way communication with the general public is important in crisis management. Even though big data technology raises some degree of privacy concerns, when used appropriately, it has the potential to assist in digital contact tracing. Conversely, using social media to spread misinformation or suppress the truth can lead to a distrust of the leadership.

#### Community Engagement and Multisectoral Actions

Having a synchronized government response alone would be insufficient to contain the spread of the virus. To effectively minimize cases, it was also crucial to have multi-sectoral contribution and have citizens make personal sacrifices for the greater public health’s good. In Thailand, daily coronavirus briefings created a general awareness of the situation’s seriousness, leading to people’s commitment towards protecting themselves and others. On the community level, Thai village leaders facilitated home quarantines and enforced mask-wearing in public spaces. On the national level, Thailand’s reopening plan was carefully constructed after consulting with public health experts and stakeholders from the public and private sectors [17].

Meanwhile, South Korea never underwent a complete lockdown of cities or provinces. School systems phased into online platforms in April of 2020 to protect the health and safety of students, teachers and their families. In general, stay-at-home campaigns were well-received by the population. South Koreans cooperated with contact tracers and abided by the government’s decisions because people had learned from past mistakes during the MERS outbreak [16]. According to a survey conducted by the Institute for Future Government, 84% of South Koreans were willing to sacrifice digital privacy for public health security [42].

In a similar fashion, the Chinese population also adhered to public health orders. When the virus became known to the public, citizens relied on social media as a medium for health intervention [43]. Many of them held fresh memory of the SARS epidemic in 2003 and were alert about a new outbreak [44]. In spite of initial government censorship, citizens tried to disseminate any available information from health-related forums to remind their friends and families of mask wearing, hand washing and social distancing [43]. Alliances were formed between manufacturing sectors to increase PPE supplies [45]. The government also allocated an additional US $1.44 billion towards the prevention effort [11].

Australia’s success in containing the initial clusters, as well as the subsequent outbreaks in Victoria and South Australia, were also attributed to the citizens’ engagement. A large majority of Australians were cooperative about following state policies, such as the mandatory 14-day hotel quarantining [21]. Compared to other Organization for Economic Co-operation and Development (OECD) countries, Australia experienced one of the least striking economic downturn during the pandemic due to generous stimulus packages aimed at promoting cash flow amongst small and medium-sized businesses [46].

In New Zealand, the leadership took a “care ethics approach” to display self-sacrificing gestures [14]. For six months, all ministers and executives working in government agencies took a 20% pay cut to stand in solidarity with those that lost their jobs or received salary cuts [47]. The leadership also inspired communitarian ethics, advocating for collective responsibility and “putting others first at the expense of short-term economic reasoning.” [14] Although the country effectively eliminated domestic transmissions, economic activities from tourism was severely impacted by national lockdowns and global travel bans [48]. In response, the Ardern administration issued fiscal stimuli, most notably a NZ $50 billion allocation towards COVID recovery fund in May of 2020 [49].

The German government also reacted to lockdown-induced economic damage generously. Instead of letting employees file for unemployment, a short-term work program called “Kurzarbeit” was initiated to let employees work fewer hours every week and still receive up to 87% of their usual payroll [50]. Even though there was a certain degree of backlash against COVID-19 safety measures and vaccinations by conspiracy theorists and anti-vaxxers, many Germans still came together to produce makeshift face coverings during times of severe mask shortages [51, 52].

While the aforementioned governments tried to balance economic gains with collective health, higher burden countries did not achieve such harmony. In Italy, for example, conflicting messages between the scientific community and the government caused civilians to undermine social distancing decrees [37]. There was substantial tension between political actors, causing closure and reopening plans to be in disarray across the nation [37]. In the US, there was a similar lack of multi-sectoral coordination and community engagement. Firstly, American polling revealed a strong partisan divide regarding the COVID-19 outbreak, suggesting that many individuals were in denial of the situation’s severity [53]. Their poor cooperation prevented the country from truly flattening the curve since spring of 2020 [27]. It was equally worrisome to observe that, after months of being on lockdown while seeing news about gun violence and police brutality, many Americans became numb towards the virus’s danger and developed a willingness to accept mass death as an inevitable part of the country’s history [27]. Lastly, since each state decided its own lockdown, curfew and reopening timeline, universities that returned to in-person learning without cautious planning suffered major outbreaks [54]. Blunt lockdown plans also escalated the national unemployment rate to 14.8% in April of 2020 [55]. And despite the passing of a US $2 trillion economic stimulus bill, small businesses were not prioritized to receive loans over large, politically-affiliated corporations [26, 56].

In Brazil, the president echoed an anti-science sentiment that polarized the society into supporters versus critics of his actions [24]. Brazilian polling showed an increasing percentage of respondents that did not intend on receiving the COVID-19 vaccine when one becomes available [25]. The nation’s flawed multisectoral actions also paralleled with those in the US. Like many American state governments, the Brazilian administration lifted restrictions and allowed for businesses to reopen before cases stabilized at a safer level. For that reason, many people began to participate in social gatherings and behaved under the assumption that they were invincible, causing cases to skyrocket again in the later months of 2020 [57]. While Brazil emerged from 2020 with the least economic recession in Latin America, emergency assistance funds were poorly allocated domestically, often going toward major firms rather than the more severely impacted small and medium-sized companies [24, 58].

#### Public Health Capacity and Response

Shortages of PPE were prevalent everywhere at the start of the pandemic as people scrambled to stockpile face coverings and hand sanitizers. With that said, a government’s capacity to adapt promptly, gather ample resources, prepare the health workforce, and provide fiscal support is key in the framework of public health capacity [59]. Evidence shows that public health capacity strongly correlates with a country’s ability to contain the spread of infectious diseases.

When the first case of COVID-19 was confirmed in South Korea, the government activated the EUA system and allocated budgets toward test kit production well before the virus reached many other countries. South Korea’s mass testing capacity and testing turnaround time were attributed to the organized collaboration between the public and private sectors [16]. The nation also pioneered the use of drive-through screening clinics, which eventually became a model to be followed by other countries around the world [60]. After the MERS epidemic in 2015, the government expanded the health workforce and increased funding from US $62 million to US $175 million, ensuring that homeless people and non-citizens in South Korea have access to essential supplies and services as well [16]. In Germany, the government also approached the pandemic with an aggressive early testing strategy. Researchers at the Charité Hospital in Germany began developing one of the first COVID-19 tests before the WHO officially declared the virus’s transmissibility [61]. Both public and private labs were mobilized to scale up the testing capacity [31]. And by February of 2020, the administration had mandated all insurance companies to pay for COVID-19 tests, promising free tests for anyone residing in Germany.

Thailand started off with a lower testing capacity relative to South Korea and Germany. There were only two laboratories conducting COVID-19 tests initially, but the number quickly grew to over 200 certified facilities that could process 10,000 to 100,000 tests per day [62]. On the community level, a group of over one million village health volunteers (VHVs) supported primary health services and educated Thai civilians on COVID-19 prevention measures [63]. With such a vast team of health promoters and rapidly improving testing capability, Thailand did not record a single local transmission between 25 May 2020 and 22 October 2020 [16]. A similar achievement was witnessed in New Zealand, where early mass testing and commitment towards a “clearly articulated elimination strategy” allowed the country to be free of domestic transmissions for over 100 days [64].

Australia was another proponent of mass testing and tried to adopt a similar response to New Zealand’s. The government allocated over AUD $750 million to support testing capacity, allowing the country to reach one of the highest tests per 1000 population in the world during the first few months of outbreak [65, 66]. In April 2020, Australian virologists expanded the scope of surveillance, allowing more people in the community to become eligible to receive the COVID-19 test [67]. Furthermore, the government spent AUD $171 million to set up and operate over 150 respiratory clinics in the countryside, intending to care for the most vulnerable populations in rural parts of Australia [66].

In China, transmissions were brought under control through batch-testing [68]. This efficient process allowed all 11 million residents in the pandemic epicenter to be tested by May, returning the region to some sense of normalcy after months of strict lockdown [68]. It is important to note that not all countries saw this level of successful containment via testing. In the small Italian town of Vò, for instance, early mass testing eradicated the virus from this town in March of 2020 [69]. For the rest of the country, however, the disease incidence already exceeded beyond the level of control [70]. And with limited contact tracing capacity, the Italian jurisdiction resorted to abrupt lockdowns that were adversely taken by the general public [69].

Similarly, the US lost crucial weeks in February of 2020, when the first few cases could have been identified and traced, because many early test kits were found to give faulty diagnoses [71]. Even though the nation eventually caught up on mass testing and was performing the second most COVID-19 tests per million amongst the countries most severely impacted by the pandemic, the feat to catch up occurred beyond the point of halting summer and winter surges [9, 26]. Interestingly, the American government spends nearly twice as much on healthcare as an average OECD country, but its healthcare workforce has been paradoxically understaffed, an issue that became magnified during periods of surging caseload [72, 73].

The Brazilian leadership was also denounced for its negligence to set up a surveillance network early on. In March of 2020, the nation’s own health minister advised against mass testing, considering it a waste of public funding [74]. By July of 2020, Brazil rolled out COVID-19 tests 10 times slower than the US. Those tests’ accuracies were also compromised by the federal government’s decision to use rapid serological tests instead of the more accurate polymerase chain reaction tests [24, 75]. Furthermore, the public health capacity in different municipalities varied greatly. A considerable number of Brazilian regions were receiving less funding from the federal government due to political tension with the leadership [76]. As the country struggled with PPE shortages, unprotected frontline health workers visiting rural areas of Brazil might have unintentionally exposed the vulnerable Indigenous populations they intended to care for with the virus [25].

### COVID-19 Deaths and Deaths per Million

There were four main indicators that explained the variability of COVID-19 deaths and deaths per million in the nine countries. They were: 1) universal health coverage, 2) medical services and hospital capacity, 3) demography and 4) burden of non-communicable diseases.

#### Universal Health Coverage

COVID-19 shed light on health inequalities and underscored the need for universal health coverage (UHC) to lessen cost barriers that hinder people’s access to lifesaving treatments during a pandemic. Thailand, for instance, is one of the few middle-income countries to have achieved universal healthcare by 2002. Thai citizens choose from one of three public health insurance options: 1) the Civil Servant Medical Benefit Scheme for civil servants and their families, 2) the Social Security Scheme for private employees and 3) the Universal Coverage Scheme. During the COVID-19 pandemic, all individuals residing in Thailand were granted access to testing and intensive care treatment at public and private facilities for free, regardless of insurance enrolment or citizenship status. Foreign patients without private insurance received equally high quality care, fully paid for by government contingency funds [17].

China is also an upper-middle-income country, and its UHC scheme has impressively covered the 1.3 billion people population since 2011 [77]. This achievement is a culmination of multiple drivers from the previous decades. Primarily, the government invested in a more comprehensive UHC scheme after the SARS outbreak exposed systemic weaknesses. Secondly, dissatisfaction amongst citizens increased as people struggled to afford their medical bills and were forced to pass up on treatments. Thirdly, with decades of economic upsurge, the Chinese government directed that fiscal capacity towards subsidizing the one billion people’s health insurance [77]. To this day, the country still faces health disparities between rural and urban areas. Nevertheless, UHC has allowed every local and the many domestic migrants to receive free COVID-19 treatments, demonstrating UHC’s necessity in effectively keeping pandemic deaths to a minimum [78].

In Germany, health insurance has been compulsory for the entire population since 2009. 90% of the population are covered under the public “statutory health insurance” (SHI), while the remaining 10% opt for “private health insurance” (PHI). The SHI requires employers to contribute towards half of the employees’ health insurance, and it offers basic coverage through any one of around 100 non-profit funds. Meanwhile, the PHI is used by self-employed individuals and high-income employees; it provides a larger selection of providers and faster access to non-emergency services [79]. In May of 2020, the German government also opened up intensive care unit (ICU) beds to patients from neighboring EU countries, on top of already providing free COVID-19 testing and treatment for anyone residing in Germany [31]. Out of the nine countries in this study, Germany has the second largest population cohort above the age of 65, meaning that more individuals are at high risk of dying from the virus [9]. However, with effective use of UHC, the country limited COVID-19 mortality amongst the elderly population early on, which explained why its CFR was much lower than those of comparable countries [31].

Australia’s UHC has been characterized as one of the most comprehensive insurance plans in the world, and its framework is comparable to the previously discussed German insurance schemes. That is, it also follows a two-tiered structure in which private health insurance works in tandem with Medicare. Most Australian citizens and permanent residents have universal health coverage through Medicare, which is tax funded. Private health insurance strives to fill those gaps within Medicare by offering faster access to non-urgent services and a larger selection of health providers that are not necessarily subsidized through Medicare; these include pharmacists, optometrists, dentists and physical therapists [80]. During the COVID-19 pandemic, treatment and testing charges were waived for every individual living in Australia, including overseas travelers who were ineligible for Medicare or had inadequate travel insurance coverage.

In New Zealand, the ideology of providing healthcare for all stemmed from the Social Security Act of 1938 [81]. Through a tax funded UHC scheme, all permanent residents have access to inpatient, outpatient, preventative, mental health, long-term and pharmaceutical services. Approximately one-third of the population purchases private insurance to expedite non-urgent treatments [81]. The nation has kept its COVID-19 death rate to a minimum due to the administration’s decision to undertake an “elimination strategy” early on [82]. The strategy includes providing free COVID-19 testing and treatment to anyone presenting symptoms, regardless of citizenship status and insurance type [83].

In South Korea, all citizens are covered under the National Health Insurance (NHI). With this centralized health system, medical resources are mobilized in a more streamlined fashion during health emergencies [16]. The NHI follows a framework similar to the German social insurance system in the sense that health insurance contribution for an employed person is proportional to income level and shared equally between the employee and employer. Meanwhile, insurance for a self-employed person is based on the individual’s income and property ownership [84]. During the COVID-19 pandemic, all South Korean citizens were eligible for free testing and treatment. The government also made it a priority to provide the same services for free to vulnerable populations, such as homeless people and immigrants without legal status [17].

Brazil also has UHC through a tax-funded system called the Sistema Único de Saúde (SUS), which was created in 1988 when the Brazilian constitution defined health as a universal right [85]. All individuals living in Brazil regardless of citizenship status are covered under this system, which entails free access to all forms of health services, namely, primary care, hospital care, outpatient specialty care, mental health care, dental care, physical therapy, optometry, and pharmaceuticals [85]. At the same time, approximately 25% of Brazilian citizens opt for employment-based private health insurance to receive care with shorter wait times [85]. The SUS has a long history of being underfunded [85]. When COVID-19 cases began to rise in Brazil, this issue became even more apparent and pertinent; while middle to high income families could afford private health insurance to receive COVID-19 treatment sooner, the larger majority of the population must use the SUS if they fall critically ill, and be faced with potential staff and supply shortages [86].

By the same token, Italy has a UHC system called the Servizio Sanitario Nazionale (SSN) that was ill-prepared to combat the COVID-19 pandemic after years of budgetary cuts [87]. The SSN was established after the Italian constitution declared health as a universal right in 1978 [88]. This tax-funded system automatically covers all citizens and foreign residents with primary care, hospice care, inpatient care, pharmaceuticals and health screening [89]. While supplementary private health insurance plays a limited role in the Italian health system, it does offer patients with more comfort and privacy in hospital settings [89]. The SSN went through significant reformations over the decades to maximize its efficiency, but interregional funding disparity remains a major challenge [89]. With the ongoing COVID-19 pandemic, it has become increasingly clear that the health inequities between affluent and impoverished communities are being exacerbated, resulting in one of the highest country death tolls in Europe [88].

Unlike most OECD countries, the US does not have universal healthcare. Instead, health coverage is offered through social insurance programs and private health insurances [90]. The former includes programs like Medicare, Medicaid, Children’s Health Insurance and the Veterans Health Administration. The latter can be accessed either through an employer-sponsored program or purchased on an individual basis [90]. Typically, employer-sponsored health insurances are purchased by businesses as part of the employees’ benefit package, and this was how approximately 55.4% of Americans were covered in 2019 [90]. The US is ranked first for healthcare expenditure per capita in the world, spending nearly twice as much as the average OECD country [91]. High costs are primarily driven by the use of advanced medical technologies, increase in chronic disease prevalence burdening Medicare, and high administrative costs that make up more than one-third of healthcare spending [92]. With that said, these systemic flaws prevented many Americans from affording basic health services, making them highly vulnerable during the COVID-19 pandemic. Without UHC, critically ill patients were not inclined to seek medical help or follow through with treatment due to fear of incurring medical debts [93]. This situation was exacerbated by the staggering unemployment rate, which caused as many as 7.7 million Americans to lose their employer-sponsored insurances by June of 2020 [94]. As of January of 2021, the US recorded the highest COVID-19 death toll in the world. In summary, having a resilient health care system centered around UHC is essential to effectively manage a pandemic and produce more favorable health outcomes.

#### Medical Services and Hospital Capacity

COVID-19 posed unprecedented challenges for medical systems globally. The need to prioritize the pandemic response inadvertently compromised non-urgent health services and raised people’s fear of acquiring the virus during clinic and pharmacy visits. This was a predicament that would inevitably increase morbidity and mortality rates in other illnesses. To combat this drawback, countries have embraced the use of video-conferencing software to enable telemedicine consultations between patients and primary care physicians [95]. Furthermore, South Korea, Thailand, Australia and the US began to offer free postal delivery of prescription drugs to minimize in-person contact [16, 17, 96, 97].

Most countries around the world experienced shortages of hospital beds during the initial surge in cases. However, a few noteworthy countries effectively expanded their medical infrastructures to curb mortalities. China, for instance, built specialty hospitals to accommodate the upsurge of cases in Wuhan [98]. Individuals who were exposed to COVID-19 or presented mild symptoms were either monitored at home or isolated at stadiums that were converted into temporary “Ark Hospitals.” [99] Meanwhile, South Korea has one of the highest numbers of beds per capita relative to other OECD nations, standing at 12.3 beds per 1000 population [100]. However, its hospital system still experienced a brief shortage of ICU beds during the Daegu outbreak in February 2020, which was later resolved by moving patients with lower risk symptoms to “residential treatment centers.” [16] Similarly, mild and asymptomatic cases in Thailand were isolated in designated dormitories such that ICU beds could remain available for patients requiring more acute care [17]. Likewise, the Australian government poured AUD $171 million into the maintenance of over 150 respiratory clinics that redirected patients with non-urgent symptoms away from the emergency rooms [66].

In Europe, Germany records the highest number of hospital beds per 1000 and ranks within the top five EU nations for the number of doctors and nurses per 1000 population. With careful stepwise planning, its hospital system gradually resumed some elective surgeries, while taking in COVID-19 patients from other EU nations [31, 101, 102]. Italy was one of the EU members that requested support after struggling with acute staff and PPE shortages [37]. In March 2020, the situation was so dire that healthcare personnel in Lombardy released video statements about their distress [37].

Brazil attempted to expand its healthcare capacity by building additional hospital wards [103]. Despite adding hospital beds at an unprecedented speed, local emergency rooms struggled to keep up with the growing hospitalization rate. In the Amazons, for instance, hospital and funeral systems were oversaturated to the point of collapsing twice in 2020. Many patients of those regions continued to die of asphyxiation due to oxygen shortages in the ICUs [104]. As of January of 2021, Brazil held the world’s second highest COVID-19 death toll, only surpassed by the US [105]. Inevitably, the distressing hospitalization rate took a toll on Brazil’s frontline medical staff as well, as the nation recorded the highest death rate of nurses infected by COVID-19 in the world [24].

Meanwhile, New York became the first COVID-19 hotspots in the US in March of 2020. Healthcare personnel were diverted to support hard-hit regions, and the Javits Center was converted into a medical facility to accommodate 2000 extra beds [26]. New York’s adversity became not only a learning opportunity, but also a grim warning for the rest of the nation to strengthen hospital capacity. In response, President Trump authorized used of the Defense Production Act to accelerate the production of ventilators [26]. With cases surging during summer and holiday months, the country faced a new challenge: hospitals had an ample reserve of available ventilators, but not nearly enough ICU doctors, respiratory therapists and pulmonologists with proper training to treat the number of critically ill patients that were being admitted [106]. Moreover, frontline health workers were also getting infected at an alarming rate. Emergency rooms turned away patients with urgent need on a daily basis, regardless of the type of illness [107]. Like in Brazil, the American health systems were being pushed to the brink of collapse.

#### Age Structure and Burden of Non-Communicable Diseases

A country’s demographic profile indicates how well its population will fare during public health crises. In this study, we compare nine countries’ age structures and NCD DALYs. The rationale is that the dual stress of population aging and comorbidities will make some countries more vulnerable than others for COVID-19 mortality [108]. For instance, Table 1 shows that Italy has the highest proportion of elders making up its population. Since older adults are more likely to develop severe symptoms with the virus, Italy ranks first in COVID-19 deaths per million out of the nine countries [109]. Following closely behind Italy are the US and Brazil. These two countries do not experience population aging to the extent of Italy [110]. Nevertheless, they both measure relatively high NCD DALYs, suggesting that a considerable proportion of their population is at risk of developing life-threatening COVID-19 symptoms compounded by comorbidities [108].

## Discussion

The epidemiology of COVID-19 is variable in different countries around the world. While the US, Italy and Brazil witnessed skyrocketing case and death counts, South Korea, China, Thailand, Australia, New Zealand and Germany experienced relatively lower disease burden. The heterogeneous outcomes can be attributed to a number of factors, which include response coordination, communication, public health capacity, universal health coverage, hospital capacity, age structure, and burden of NCDs. Understanding how these factors play a role in pandemic responses allows hard hit countries to learn from lower burden countries and become better prepared for re-emerging outbreaks.

Over the course of the pandemic, governments around the world increased the rigor of their intervention plans in hopes of containing the virus. Our study shows that countries governed by leaders who took rapid actions, provided generous welfare support, and synchronized policies with scientific observations were most effective at curtailing case counts. This is consistent with other study’s findings, which praise countries like New Zealand for its early lockdown. Similarly, Singapore has been commended for learning from past disease outbreaks and establishing a multi-ministry COVID-19 taskforce before its first confirmed case [111]. Other researchers also agree that, although lockdowns and closures should be urgently implemented to curb transmissions, building healthy behaviors on the individual level and fostering compliance on the community level are just as fundamental, which cannot be accomplished unless leaders gain the trust of the general public through transparent communication [112]. In addition, our study concluded the importance of having a strong public health system in order to effectively locate confirmed cases and halt further infections. This idea is shared by the WHO, which has openly criticized countries that did not prioritize testing and tracing early on [2]. Countries like the UK and Haiti, for instance, did not scale up their surveillance networks quickly enough, and they ended up with uncontrollable spread of the virus caused by individuals who were not eligible to be tested from the start [2, 113]. Interestingly, a study on serologic testing found that a small percentage of blood donations in three states from Western US showed COVID-19 antibodies in mid-December of 2019 [114]. This finding not only suggests that American transmission commenced earlier than people originally thought, but also implicates the possibility of using blood donations to perform population-based prevalence studies during future epidemics [114].

Our study reveals that numerous high-income and upper-middle-income countries have UHC systems, but years of budgetary cuts have left them ill-equipped to fight the pandemic. This phenomenon resonates with other literature’s findings, which underscore the importance of adequate funding for UHC to protect the most vulnerable populations from catastrophic health expenditures [115]. UHC undoubtedly comes with inherent constraints as well, such as longer wait times and less access to advanced biotechnology [116]. Nevertheless, ensuring financial protection and eliminating inequities are irreplaceable themes of the Sustainable Development Goals [117]. In addition to using UHC to overcome health inequalities, other research have concluded that the ability to expand medical infrastructures before case surges is key in curtailing COVID-19 deaths. One study mentions that the UK entered the pandemic with a low number of beds and physicians per capita relative to other OECD countries [118]. A year after the pandemic’s onset, the nation was reporting similar deaths per million as Italy [9]. Finally, we concluded that population aging and chronic disease burdens exposed underprepared countries to high mortality rates. Of all the NCDs, the most common comorbidities associated with unfavorable COVID-19 outcomes are hypertension, chronic cardiovascular disease, diabetes and chronic cerebrovascular disease [119]. An article in the British Medical Journal agrees that, despite the overall CFR of COVID-19 being low, older individuals with those underlying NCDs are still much more likely to suffer from severe symptoms leading to mortality [120].

The results from this study are subject to certain limitations. Firstly, there was likely an underestimation in the number of COVID-19 cases each day because some countries had insufficient testing resources or showed negligence towards reporting accurate diagnostic records. Secondly, the death count was possibly skewed because every country followed its own protocol to define mortality caused by COVID-19 infection; some countries only examined clinical presentations, while others relied on positive laboratory tests to confirm COVID-19 deaths [121]. Public health authorities have reasoned that heterogeneity in reporting guidelines is likely to be politically motivated [122].

At the same time, other caveats exist when comparing COVID-19 statistics across different countries. For one, patients with chronic diseases made less frequent hospital visits during the pandemic, hindering them from receiving proper care for otherwise curable illnesses. In countries with high disease burden, emergency services were overwhelmed with COVID-19 patients, making first responders unavailable to respond to other common types of life-threatening events. Conversely, stay-at-home orders decreased traveling and likely reduced mortality caused by traffic accidents. All in all, given the variation in reporting mortality statistics and the underestimation of cases, cross-country comparisons of COVID-19 fatality rates should be done with caution.

## Conclusion

The COVID-19 pandemic has variable effects across countries due to differences in their vulnerability, preparedness and response. Our study highlights key action areas in crisis management and demonstrates their interactions to affect caseload and mortality. For instance, a strong testing capacity is only effective when combined with thorough contact-tracing. Behavioral change and community engagement require political leaders to not only earn the people’s trust through transparent communication, but also put trust in the scientific community themselves. Investing in hospital capacity in the long-term proves to be lifesaving when cases do surge out of control. While we are still far from achieving UHC globally, allocating generous funds toward welfare programs and taking gradual steps to eliminate health inequalities show promise in decreasing comorbidities, increasing life expectancies and minimizing COVID-19 mortalities. To conclude, our research offers valuable lessons to prevent and control COVID-19, as well as to prepare for future epidemics if countries are proactively adapting and implementing them.

## Data Availability

The datasets supporting the analyses of this study are publicly available on the *Our World in Data* website (https://ourworldindata.org/coronavirus-data) and the *World Bank* website (https://data.worldbank.org/indicator/NY.GDP.PCAP.CD). The qualitative secondary data used to support the study’s findings are publicly available and cited in the References.

## Abbreviations

COVID-19: Coronavirus disease of 2019
WHO: World Health Organization
US: United States
EU: European Union
CFR: Case fatality rate
GNI: Gross national income
NCD: Non-communicable disease
DALYs: Disability-adjusted life years
SARS: Severe acute respiratory syndrome
MERS: Middle East Respiratory Syndrome
EUA: Emergency Use Authorization
RKI: Robert Koch Institute
PPE: Personal protective equipment
OECD: Organization for Economic Co-operation and Development
VHV: Village health volunteer
UK: United Kingdom
UHC: Universal health coverage
SHI: Statutory health insurance
PHI: Private health insurance
ICU: Intensive care unit
NHI: National Health Insurance
SUS: Sistema Único de Saúde
SSN: Servizio Sanitario Nazionale
